# Orbital teratoma in the foetus: a rare case without proptosis

**DOI:** 10.1186/s12886-020-01681-w

**Published:** 2020-10-19

**Authors:** Xi Chen, Jiaxiang Yang, Guannan He, Chunlan Cheng, Chunguo Zhang, Hongli Wang, Lihong He, Zhirong Yang, Li Chen, Jing Zhao

**Affiliations:** 1 Department of Ultrasound, Sichuan Provincial Hospital for Women and Children, No.290 Sha Yan Xi Er Street, Chengdu City, Sichuan Province China; 2 Department of Clinical Laboratory, Sichuan Provincial Hospital for Women and Children, Chengdu, Sichuan China; 3 Department of Pathology, Sichuan Provincial Hospital for Women and Children, Chengdu, Sichuan China; 4 Department of Pathology, People’s Hospital of DeYang City, DeYang City, Sichuan China

**Keywords:** Orbital teratoma, Ultrasound, Proptosis, Foetal

## Abstract

**Background:**

Congenital orbital teratoma is relatively rare, and few reports of prenatal ultrasound findings in such cases have been published.

**Case presentation:**

A rare case of congenital orbital teratoma at 24 + 2 weeks of gestation was previously diagnosed as microphthalmia, noting how orbital teratoma without proptosis is different from microphthalmia, retinoblastoma and intracranial teratoma. Ultrasound examination, analysis of gross specimens, and histopathological evaluation confirmed the diagnosis of orbital teratoma.

**Conclusion:**

Prenatal ultrasound examination is useful for diagnosis and differential diagnosis of congenital orbital teratoma.

## Background

Congenital orbital teratoma is relatively rare. These lesions are classified as mature or immature teratomas based on their degree of cellular differentiation. Orbital teratomas have been detected at birth or, more commonly, early in life due to clinical proptosis [1]. Nevertheless, foetal information is limited. Here, we report a prenatal case of mature orbital teratoma without proptosis at 24 + 2 weeks of gestation, with an initial diagnosis of microphthalmia. We present the ultrasound characteristics, gross specimens, and histopathological appearance.

## Case presentation

**Fig. 1:**
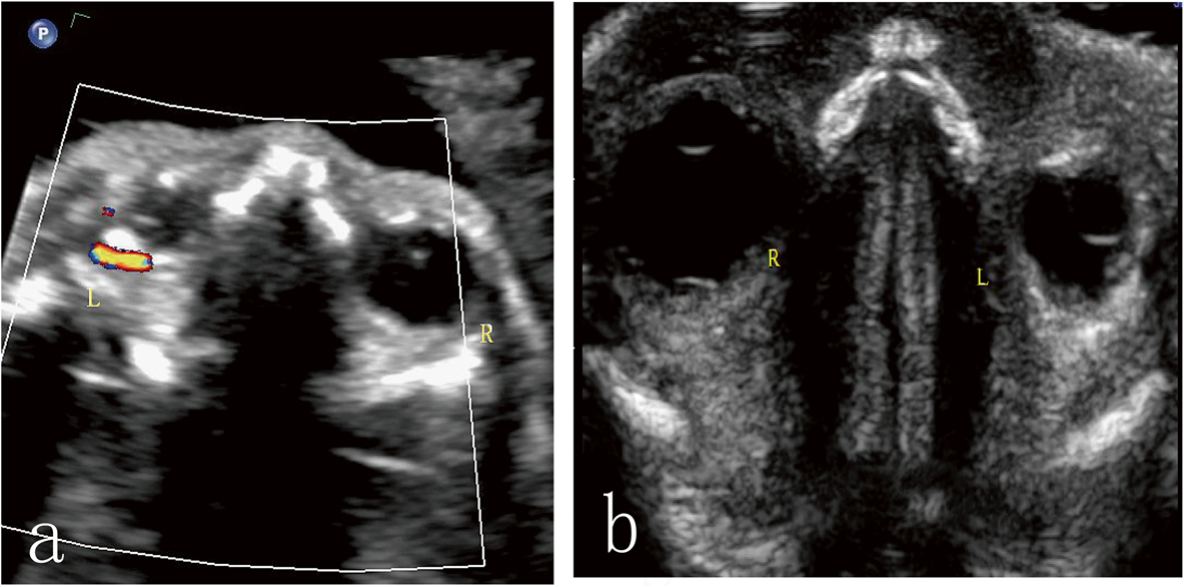
Prenatal sonography in the transverse plane at 24 + 1 weeks of gestation. The left eye globe was much smaller than the right eye globe. Colour Doppler imaging showed blood flow signals in the lesion (**a**). Using high-frequency ultrasound, a hyperechoic band was observed in the retro-orbital space (**b**)

A 25-year-old pregnant woman, gravida 1, para 0, at 24 + 2 weeks of gestation was referred to our department for prenatal ultrasound examination. Prenatal ultrasound examination revealed that the foetus’ eyes were asymmetrical. The right and left eye globes measured 10.9 × 8.3 mm and 6.8 × 6.0 mm, respectively. The mean normal foetal orbital diameter is 11.0 mm at 24 weeks of gestation [2]. An initial diagnosis of microphthalmia was made. A hyperechoic lesion was detected in the left retro-orbital space. The lesion was crescent shaped, with a maximum thickness of about 1.6 mm (Fig. 1b); it was confined to the orbit and showed no proptosis. No shadowing calcifications were detected. Colour Doppler ultrasound examination demonstrated significant angiogenesis in the lesion (Fig. 1a). Prenatal ultrasound examinations showed no structural abnormalities other than asymmetry of the eyes, and the intracranial structure did not appear to be deformed. The head circumference, abdominal circumference and femur length were consistent with the gestational age.

After consultation, the patient and her husband indicated that they wanted to induce labour. Autopsy demonstrated a normal facial appearance (image could be available upon reasonable request from the corresponding author).

**Fig. 2:**
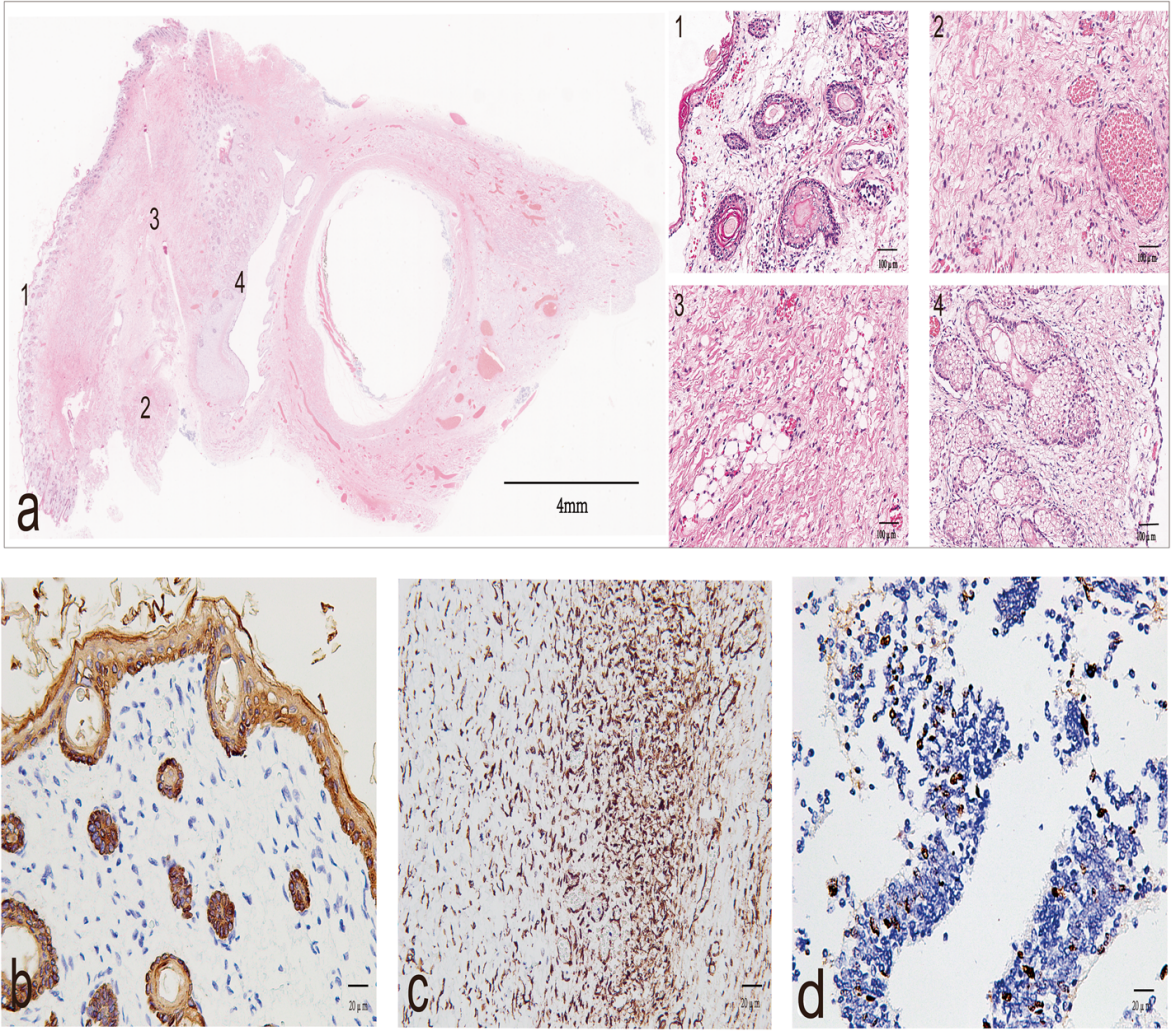
Histopathology of the tumour specimen. The vitreum is on the right of **a**. Islands of tumour tissue were detected in the retro-orbital space. The tumour contained skin and skin appendages (1), vascular tissue cells (2), fatty tissue (3), and differentiated mature glands (4). Strong, diffuse membrane immunohistochemical staining for CK indicated skin and skin appendages (**b**) and immunostaining for vimentin confirmed differentiated mature glands (**c**). The Ki-67 nuclear staining index was approximately 10% (**d**). Scale bars: Fig. a, 4 mm; Fig. a1–a4, 100 μm; b, c, d, 20 μm

Histopathological examination showed a mature orbital teratoma in the retro-orbital space (Fig. 2). The teratoma was predominantly benign, with mature tissue originating in the ectoderm (epidermis and its derivatives), mesoderm (fatty tissue, blood vessels), and endoderm (gastrointestinal epithelium) (Fig. 2a). On immunohistochemical examination, the gland origin was confirmed by cytokeratin (CK) immunopositivity (Fig. 2b). The mesenchymal tissue showed diffuse and strong positive immunostaining for vimentin (Fig. 2c). In the mature parts of the teratoma, the cell proliferation rate determined using Ki-67 as a marker was about 10%, similarly to the level of normal tissue (Fig. 2d). Glypican-3 (GPC-3) and neuron specific enolase (NSE) staining were negative.

## Discussion and conclusion

Congenital orbital teratomas are quite rare. They are often reported in female infants and children with characteristic proptosis [1–3]. However, information on foetal cases is very limited (see Table 1). In our case, orbital teratoma was detected at 24 + 2 weeks of gestation, and the lesion was confined to the orbit without proptosis. To our knowledge, this is the first report of a foetal orbital teratoma without proptosis. Anami et al. [4] reported a large orbital teratoma with proptosis at 27 weeks and intrauterine foetal death at 32 weeks of gestation. We suspect that the absence of proptosis in our case was because the disease was in its early stage. Although orbital teratoma is often associated with rapid enlargement soon after birth [3, 5], the absence of proptosis can lead to confusion and a delay in diagnosis. Therefore, screening of the retro-orbital space is essential for diagnosis of orbital teratoma, especially in cases without proptosis.

**Table 1:** Summary of five recent case reports about prenatal imaging of orbital teratoma

Study (Author/year)	Gestational age (weeks)	Sex	Proptosis	Left/right/bilateral	MRI/US
Anami A/2012 [4]	27	Male	yes	left	US
Herman TE/2009 [6]	38	Female	yes	right	MRI
Moon YJ/2018 [7]	17	Female	Yes	left	US
Mamalis N/1985 [8]	28	Male	yes	left	US
More GHM /2019 [9]	35	Male	Yes	right	MRI

Firstly, as this case had no proptosis and asymmetrical eyes, it was initially misdiagnosed as microphthalmia, the most likely cause of small eyes. Orbital teratoma and microphthalmia can be distinguished by three differences. First, microphthalmia is more commonly bilateral. The exception appears to be isolated microphthalmia, which is usually unilateral. Microphthalmia shows no predominance with regards to gender, while orbital teratomas are usually unilateral and more often seen in females. Second, microphthalmia results in a small orbit volume compared to age-matched controls [10], whereas orbital teratoma results in enlarged, remodelled, or destroyed orbit [11]. Finally, in microphthalmia, ultrasound examination does not show blood flow signals in the orbit. In contrast, colour Doppler ultrasound shows a clear blood flow signal in the lesion in the present case of orbital teratoma [4]. In microphthalmia patients, the potential for visual development depends on the degree of retinal development and other ocular characteristics. Therapy aims to maximise existing vision and enhance cosmetic appearance rather than improve sight [10]. In orbital teratoma patients, timely and accurate diagnosis enables early surgical resection of the orbital teratoma with satisfactory cosmetic results, consisting of preservation of the eyeball and visual function [12]. The prenatal orbital diagnosis of teratoma is important to allow timely counselling of the parents and as an aid in obstetric decision making.

In addition, retinoblastoma as the most common intraocular malignancy of infancy and childhood demonstrates a mass more echogenic than the vitreous, with fine calcifications by ultrasonography. Retinal detachment may also be observed in exophytic forms [13]. While the orbital was always in normal values scale without mass effecting the retro-orbital space [14, 15].

Finally, intracranial teratoma invading the orbit may not cause proptosis. Arslan et al. [16] reported a large intracranial immature teratoma extending into the retro-orbital space. In the present case, the lesion was located in the retro-orbital space, and the foetal intracranial structure was normal.

In conclusion, screening of the retro-orbital space is essential for diagnosis of orbital teratoma. And it is important to perform colour Doppler ultrasound and screening of intracranial structures in cases of abnormal orbital lesions. These findings suggest that prenatal ultrasound examination should play a critical role in the diagnosis and differential diagnosis of orbital teratoma.

## Data Availability

The data and images used or analysed during the current study available from the corresponding author on reasonable request.

## Abbreviations

CK: cytokeratin
GPC-3: Glypican-3
NSE: neuron specific enolase
MRI: nuclear magnetic resonance
US: ultrasonography
